# Use of Favipiravir for the Treatment of Coronavirus Disease 2019 in the Setting of Hospitel

**DOI:** 10.1155/2022/3098527

**Published:** 2022-03-29

**Authors:** Bhitta Surapat, Warissa Kobpetchyok, Sasisopin Kiertiburanakul, Vanlapa Arnuntasupakul

**Affiliations:** ^1^Department of Pharmacy, Faculty of Medicine Ramathibodi Hospital, Mahidol University, Bangkok, Thailand; ^2^Division of Infectious Disease, Department of Medicine, Faculty of Medicine, Ramathibodi Hospital, Mahidol University, Bangkok, Thailand; ^3^Department of Anesthesiology, Faculty of Medicine Ramathibodi Hospital, Mahidol University, Bangkok, Thailand

## Abstract

**Objective:**

In a setting with a limited capacity for hospitalization, “hospitels” have been developed by using hotels as extension healthcare facilities for patients with mild illness. This study examined the clinical evidence of patients with coronavirus disease 2019 (COVID-19) who were treated with favipiravir, the main medication for treating COVID-19, in the hospitel setting in Thailand.

**Methods:**

We retrospectively collected demographic and clinical information, medication treatment, and outcome data for all patients who received favipiravir for COVID-19 during admission to a hospitel from April 27, 2021, to July 2, 2021. Risk factors for adults who could not complete treatment in a hospitel and who required hospitel transfer were analyzed.

**Results:**

In total, 421 patients were included in the study. Most patients (94.5%) received favipiravir to treat COVID-19 pneumonia. Adjunctive corticosteroids were prescribed to 42.3% of patients. Concerning the treatment outcome, 83.6% of patients completed treatment at a hospitel, and only two deaths occurred. No serious adverse drug reactions were observed. On multivariate analysis, age (odds ratio (OR) = 1.06; 95% confidence interval (CI) = 1.02–1.10, *P*=0.002), dyspnea (OR = 2.84; 95% CI = 1.25–6.44, *P*=0.013), loss of taste (OR = 107.63; 95% CI = 1.24–9337.39, *P*=0.040), corticosteroid use (OR = 12.56; 95% CI = 3.65–43.18, *P* < 0.001), and an extended duration of favipiravir use (OR = 16.91; 95% CI = 7.29–39.24, *P* < 0.001) were associated with a higher risk of hospitel transfer.

**Conclusions:**

Low rates of hospitel transfer and mortality were observed in mild-to-moderate COVID-19 patients treated with favipiravir at hospitel. Caution might be required in elderly patients, patients with dyspnea or a loss of taste, and patients receiving a 10-day course of favipiravir or adjunctive corticosteroids because these patients might require further management in the hospitel.

## 1. Introduction

The global pandemic caused by coronavirus disease 2019 (COVID-19) has challenged the global healthcare system. Initially, little was known about the disease, and patients were usually admitted to a hospitel and closely monitored to ensure timely and appropriate management [1]. Hospitalized patients would also benefit in terms of infection control. Later, patients with mild-to-moderate disease received only symptomatic treatment, and hospitel admission was not required. Patients at high risk of disease progression could also be managed as ambulatory patients with anti-SAR-CoV-2 monoclonal antibodies [2]. However, this therapeutic option is not available globally.

In Thailand, the third wave of the COVID-19 outbreak started in April 2021. Patients would be considered mild if they were asymptomatic or had mild symptoms without dyspnea or abnormal chest X-ray. Patients with evidence of abnormal chest X-ray were considered moderate disease, and if they further had dyspnea or resting oxygen saturation of less than 93% requiring oxygen supplementation, they would be classified as severe disease [3]. According to the national policy, all patients with COVID-19 must be admitted to a hospitel [3]. However, as the number of patients rapidly increased, hospitels became overwhelmed. Alternative healthcare facilities were created using hotels, so-called “hospitels,” to accommodate patients with asymptomatic or mild-to-moderate disease who were at low risk for developing severe disease. Telemedicine is used during patients' hospitel admission. Patients are provided medications and monitored by healthcare workers until discharge [4].

Favipiravir is a purine nucleic acid analog which act as an RNA polymerase inhibitor. It is activated into phosphoribosylated form (favipiravir-RTP) and inhibit viral, including SARS-CoV-2, RNA polymerase activity. Favipiravir is available in oral form with excellent bioavailability. The drug is metabolized by aldehyde oxidase and xanthine oxidase and mainly excreted in urine [5]. Overall safety profile is good with some concerns of gastrointestinal side effects and hyperuricemia [6]. Favipiravir was mainly used to treat COVID-19 during the first and second waves of infection in Thailand [7, 8]. In the current wave of infection, the national treatment guideline published on April 17, 2021, recommended a 5 or 10-day course of favipiravir in patients with COVID-19 pneumonia and patients with symptomatic COVID-19 and risk factors for severe disease [9]. For patients with symptomatic COVID-19 without pneumonia or risk factors for severe disease, favipiravir might also be considered according to the physician's discretion on a case-by-case basis. Corticosteroids should be prescribed to patients with resting oxygen saturation ≤96% or patients with exercise-induced hypoxia [9]. A meta-analysis of clinical trials described the benefit of favipiravir in terms of viral clearance and clinical improvement, but the benefit concerning mortality and mechanical ventilation remains unclear [10, 11]. However, all studies were conducted in patients who were admitted to a hospitel, and there are no data on favipiravir treatment of patients in other settings. We explored the effectiveness of favipiravir in this new setting of hospitel.

## 2. Materials and Methods

### 2.1. Setting

Ramathibodi Hospital is a 1200-bed university hospitel in Bangkok, Thailand. During the COVID-19 outbreak in 2021, hospitel beds were reserved for patients with moderate-to-severe COVID-19. Asymptomatic patients or patients with mild disease were admitted to one of two hospitels (100 and 400 beds, respectively), which served as extension healthcare facilities. Patients with resting oxygen saturation of less than 93% or patients who were clinically unstable or who had uncontrolled underlying diseases were not admitted to hospitels. For admission, patients must also be able to communicate via phone call/video call or use smartphone applications.

During hospitel admission, patients monitored temperature and blood oxygenation themselves at least two times daily using a thermometer and fingertip pulse oximeter. An exertional desaturation test was performed twice daily. Chest X-ray was routinely checked on admission on days of illness (DOI) 1–3, 5–7, and 10–12 or on day 5 of favipiravir treatment. A blood test or laboratory monitoring was not available at hospitels. Patients were monitored closely by physicians and nurses using telemonitoring via phone or video calls. Patients remained in the hospitel until discharge on DOI 10 or 14 depending on symptoms, the severity of COVID-19, and the presence of comorbid diseases. If the patients' clinical status worsened such as tachypnea, alteration of consciousness, or hypoxia requiring supplemental oxygen therapy, they were transferred to a hospitel.

All patients were provided supportive medications such as paracetamol, acetylcysteine, dextromethorphan, and chlorpheniramine as needed. Favipiravir was only prescribed according to the national treatment guidelines following an infectious disease specialist consultation [9]. The dosage regimen of favipiravir was 1800 mg twice daily on day 1 and 800 mg twice daily thereafter. For patients weighing more than 90 kg, the dose was increased to 2400 mg twice daily on day 1 and 1000 mg twice daily thereafter. For pediatric patients, a dose of 30 mg/kg twice daily on day 1 and 10 mg/kg twice daily thereafter was recommended. Patients were advised to self-administer favipiravir for five days. The duration of treatment was extended to 10 days in some cases per the physician's judgment. Pharmacists and nurses monitored medication adherence via phone calls. Adverse drug reactions were evaluated by physicians along with pharmacist consultation.

### 2.2. Study Design and Data Collection

This was a retrospective study to report clinical experience of favipiravir use at hospitel. All patients who received favipiravir since April 27, 2021, when the chest X-ray categorical assessment scheme was implemented (Table 1) [12], were included in the study. Patients' demographics and treatments were collected. Each patient's clinical presentation was recorded on the first day of favipiravir therapy. Treatment completion at hospitels, hospitel transfer, and mortality were recorded as outcomes. The study period was planned until the end of July 2021. However, earlier in July 2021, the number of patients with severe COVID-19 who required hospitalization substantially increased. Some patients could not be transferred to hospitels because of the limited capacity. Oxygen supplementation was initiated in some patients at hospitels. Later, the national treatment guidelines changed to permit favipiravir treatment to start earlier in all patients with mild symptomatic COVID-19 [13]. The study was therefore terminated early on July 2, 2021. This study was approved by the Committee on Human Rights related to research involving human subjects, Faculty of Medicine Ramathibodi Hospital, Mahidol University (MURA2021/480).

### 2.3. Statistical Analysis

Descriptive statistics were used to report patients' demographic data, clinical presentations, treatments, and outcomes. For adults, data were compared between patients who completed treatment at a hospitel and those who required hospitel transfer. Variables significant at *P* < 0.1 according to univariate analysis were included in multivariate logistic regression analysis to identify factors associated with patient hospitel transfer. *P* ≤ 0.05 was considered statistically significant. All statistical analyses were performed using IBM^®^ SPSS^®^ Statistics version 18 (IBM, Armonk, NY, USA).

## 3. Results

During the study period, 1683 patients with COVID-19 were admitted to a hospitel. Favipiravir was prescribed to 421 patients. Most patients were adults with no underlying diseases who presented with upper respiratory tract infection symptoms. Upon follow-up, the chest X-ray findings were abnormal in 94.1% patients, and 94.5% of patients were diagnosed with pneumonia. The severity of pneumonia was classified into three categories: pneumonia, pneumonia with hypoxia, and pneumonia with the progression of infiltrates. A more severe form of pneumonia with hypoxia or progression of infiltrates was observed in 37.4% of adult patients with pneumonia. Approximately half of the patients started favipiravir on the day of admission. Adjunctive corticosteroids, mainly dexamethasone, were used in 42.28% of patients. Eventually, all 421 patients completed their courses of favipiravir without early discontinuation, and 83.61% of them completed the treatment at a hospitel. Few adverse drug reactions were observed. Most patients were discharged, as only two patients died after transfer to a hospitel. Information on patients' demographics, clinical presentation, treatments, and outcomes and the indication for favipiravir is given in Tables 2–4.

Characteristics were compared between adult patients who completed treatment at a hospitel and those who required hospitel transfer. Age, dyspnea, loss of taste, an extended duration of favipiravir therapy, and steroid use significantly increased the risk of hospitel transfer (Table 5).

## 4. Discussion

In the hospitel setting, in which patients were considered to have nonsevere disease, pneumonia can be detected upon chest X-ray follow-up. Similar to the report of a patient with “walking pneumonia” who displayed only mild symptoms [14], many patients in this study were sufficiently stable for hospitel admission, but pneumonia was diagnosed upon admission, followed by favipiravir treatment. Concerning the treatment of mild-to-moderate COVID-19, there is insufficient evidence regarding the benefit of antiviral therapies. The US National Institutes of Health and the Infectious Diseases Society of America guidelines made no recommendation about antiviral use in such patients. Only remdesivir might be considered in patients at high risk of disease progression [2, 15]. However, this drug was not widely available in Thailand, and intravenous drug administration was not possible in the hospitel setting.

During the first and second waves of the COVID-19 pandemic in Thailand, the benefit of favipiravir was promising. A multicenter study of 63 patients who received favipiravir for COVID-19 revealed a clinical improvement rate of 90.5% and mortality rate of 4.8% on day 28 [7]. Another preliminary study reported the effectiveness of favipiravir in 37 patients with COVID-19 pneumonia, as 95% of patients were discharged after completely recovering [8]. However, several treatments including antimalarials and protease inhibitors were concomitantly used during that period, and thus, the efficacy of favipiravir could not be clearly established. In addition, the number of participants was small, and patients with various disease severities, including those with severe disease requiring mechanical ventilation, were included. The severity of patients in our study was similar to that of one study comparing the efficacy of favipiravir and arbidol. Among 98 patients with moderate COVID-19 pneumonia receiving favipiravir, the clinical recovery rate on day 7 was 71.43%, with no fatalities observed [16]. Note that other antiviral treatments were also allowed in this previous study. In one report of patients with similar disease severity, patients were not permitted to use other antivirals or antimalarials. This study was a randomized clinical trial of favipiravir versus standard of care. In an interim analysis, most patients had moderate COVID-19 pneumonia without requiring oxygen therapy. Patients receiving favipiravir (*n* = 40) exhibited higher viral clearance rates on day 5 than patients receiving standard of care (*n* = 20) [17]. This might explain why most patients in our study could complete treatment without hospitel transfer, eventhough pneumonia was a major indication, and patients with pneumonia with hypoxia or progression of infiltrates were also included in our study.

To identify patients at risk for hospitel transfer, some significant factors were found in multivariate analysis in the present study. Concerning patient baseline demographics, only age was found to increase the risk. This is consistent with many reports that aging was related to hospitalization [18–22]. Impairment of the immune response and decreases of the physiological reserve in the respiratory system in elderly people could lead to severe COVID-19 [23]. Regarding the clinical presentations that could lead to hospitel transfer, dyspnea and loss of taste were identified. Dyspnea or shortness of breath has been illustrated to increase the risk of hospitalization and severe disease [24–26]. However, little is known about the correlation between the loss of taste and hospitalization or disease severity [27]. Meanwhile, one study reported that this symptom was related to nonhospitalization [25]. Another study found that a composite of hyposmia, anosmia, and dysgeusia was associated with lower disease severity [25, 28]. Thus, the association of loss of taste with COVID-19 severity requires further research for clarification. In terms of treatments, our study demonstrated that the use of adjunctive corticosteroids and a 10-day course of favipiravir were associated with hospitel transfer. This could reflect the disease severity of patients because corticosteroids were recommended for the treatment of hospitalized patients who required supplemental oxygen [2]. In addition, prolonged antiviral therapy might indicate the lack of an adequate clinical response, and thus, further management in the hospitel might be required.

Our study had several limitations. Some data of symptoms were subjective and difficult to obtain in pediatric patients. Low number of pediatric patients were included and risk of hospitel transfer was only identified in adult patients. Because of the single-center, observational design of the study with no comparator because most patients received favipiravir according to the national guideline, the efficacy of favipiravir could not be concluded. Due to the limitations of telemedicine, other outcomes such as clinical improvement or recovery time were difficult to evaluate. Regarding safety, only a few adverse drug reactions reported by the patients were recorded because no intensive monitoring such as physical examinations or laboratory tests was performed. However, to the best of our knowledge, our study provided the first clinical evidence of favipiravir treatment in a setting outside the hospitel. Compared to previous reports, this study included a large patient cohort. With the low rates of hospitel transfer and mortality, the role of favipiravir for the management of COVID-19 pneumonia is promising.

## 5. Conclusion

In conclusion, this study illustrated that patients with mild-to-moderate COVID-19, including patients with pneumonia, might be considered favipiravir treatment at hospitels. Close monitoring is still required, especially in patients at risk of hospitel transfer, such as elderly patients, patients presenting with dyspnea or loss of taste, and patients requiring corticosteroids or an extended duration of favipiravir treatment (10 days). The results from this study might be further applied in patient management in a setting with limited resources for hospitalization.

## Figures and Tables

**Table 1:** Chest X-ray categorical assessment scheme.

Chest X-ray category	Description
Category 1	Normal chest X-ray or no abnormality detected

Category 2	Minor abnormalities unrelated to COVID-19 pneumonia (i) Anatomical variation, including breast implants and scoliosis (ii) Features favoring technical issues (e.g., suboptimal inspiration and off-center exposure) but not affecting film interpretation (iii) Irrelevant abnormalities, e.g., old tuberculosis, mild cardiomegaly, and aortic atherosclerosis

Category C	Low probability of or atypical for COVID-19 pneumonia, but with other clinically significant diseases requiring clinical correlation and further management (i) Other clinically significant diseases, e.g., bacterial pneumonia, active TB, congestive heart failure, pneumothorax, pleural effusion, and malignancy

Category 3	Equivocal/unsure/indeterminate for COVID-19 pneumonia (i) Some features (e.g., subtle, poorly defined opacities) that can be attributable to early/mild/atypical COVID-19 pneumonia or other causes (e.g., pseudolesions and other diseases) requiring clinical correlation and follow-up or repeated chest X-ray

Category 4	Suspicious for early/mild COVID-19 pneumonia (i) Single or multifocal unilateral poorly defined ground-glass opacities

Category 5	Typical for COVID-19 pneumonia (i) Multifocal bilateral peripheral opacities or opacities with rounded morphology

Adapted from [12].

**Table 2:** Baseline demographics.

Characteristics		Adult (*n* = 393)	Pediatric (*n* = 28)
Age		47 years (18–73 years)	9 years (5 months–17 years)

Gender	Male	147 (37.4%)	9 (32.1%)
	Female	246 (62.6%)	19 (67.9%)

Weight (kg)		62.9 (38.0–106.9)	28.0 (3.8–85.4)
Height/length (cm)		160.0 (135.0–188.0)	131.8 (64.0–176.0)^†^
Body mass index (kg/m^2^)		24.3 (15.8–38.3)	18.4 (12.4–30.1)^†^
Obesity		38 (9.7%)	1 (3.6%)

Underlying diseases	None	256 (65.1%)	23 (82.1%)
	Hypertension	66 (16.8%)	0 (0.0%)
	Dyslipidemia	30 (7.6%)	0 (0.0%)
	Diabetes mellitus	24 (6.1%)	0 (0.0%)
	Allergy	18 (4.6%)	1 (3.6%)
	Musculoskeletal disease	9 (2.3%)	0 (0.0%)
	Digestive disease	8 (2.0%)	0 (0.0%)
	HIV	7 (1.8%)	0 (0.0%)
	Neurological disease	10 (2.5%)	0 (0.0%)
	Liver disease	5 (1.3%)	1 (3.6%)
	Malignancy	4 (1.0%)	0 (0.0%)
	Heart disease	6 (1.5%)	0 (0.0%)
	Psychological disease	6 (1.5%)	0 (0.0%)
	Lung disease	4 (1.0%)	1 (3.6%)
	Thalassemia	5 (1.3%)	2 (7.1%)
	Thyroid disease	4 (1.0%)	0 (0.0%)
	Others	Benign prostatic hypertrophy 1 (0.3%), mental retardation 1 (0.3%), polycystic ovary syndrome 1 (0.3%), and pregnancy 1 (0.3%)	Biliary atresia necessitating liver transplantation 1 (3.6%)

^†^Data for height and body mass index were available for 22 pediatric patients. Data were presented as frequency (percent) except for age, weight, height, and body mass index, which were presented as median (minimum–maximum).

**Table 3:** Clinical presentations and indications of favipiravir.

Characteristics		Adult (*n* = 393)	Pediatric (*n* = 28)
Day of illness		6 (1–17)	5 (1–15)

Symptoms	Fever	158 (40.2%)	7 (25.0%)
	Cough	247 (62.8%)	14 (50.0%)
	Oxygen desaturation	131 (33.3%)	3 (10.7%)
	Dyspnea	102 (26.0%)	1 (3.6%)
	Sore throat	86 (21.9%)	5 (17.9%)
	Rhinorrhea	58 (14.8%)	6 (21.4%)
	Productive sputum	34 (8.7%)	0 (0.0%)
	Myalgia	28 (7.1%)	0 (0.0%)
	Diarrhea	22 (5.6%)	0 (0.0%)
	Anosmia	19 (4.8%)	0 (0.0%)
	Headache	18 (4.6%)	1 (3.6%)
	Dizziness	12 (3.1%)	0 (0.0%)
	Malaise	9 (2.3%)	0 (0.0%)
	Nasal congestion	8 (2.0%)	1 (3.6%)
	Loss of taste	6 (1.5%)	0 (0.0%)
	Chest pain	4 (1.0%)	0 (0.0%)
	Nausea	4 (1.0%)	1 (3.6%)
	Others	Back pain 3 (0.8%), loss of appetite 3 (0.8%), conjunctivitis 2 (0.5%), chill 1 (0.3%), eye pain 1 (0.3%), hoarseness 1 (0.3%), rash 1 (0.3%), and stomachache 1 (0.3%)	0 (0.0%)

Asymptomatic patients		55 (14.0%)	7 (25.0%)
Chest X-ray category	1	5 (1.3%)	7 (25.0%)
	2	7 (1.8%)	2 (7.1%)
	C	4 (1.0%)	0 (0.0%)
	3	153 (38.9%)	14 (50.0%)
	4	112 (28.5%)	3 (10.7%)
	5	112 (28.5%)	2 (7.1%)

Indications of favipiravir	Asymptomatic/mild symptoms without risk factors	5 (1.3%)	3 (10.7%)
	Mild symptom with risk factors	8 (2.0%)	7 (25.0%)
	Pneumonia	238 (60.6%)	17 (60.7%)
	Pneumonia with hypoxia	114 (29.0%)	1 (3.6%)
	Pneumonia with progression of infiltrates	28 (7.1%)	0 (0.0%)

Data were presented as frequency (percent) except for the day of illness, which was presented as median (minimum–maximum).

**Table 4:** Treatments and outcomes.

Characteristics		Adult (*n* = 393)	Pediatric (*n* = 28)
Start favipiravir on admission		201 (51.1%)	14 (50.0%)

Duration of favipiravir	5 days	296 (75.3)	27 (96.4%)
	10 days	97 (24.7%)	1 (3.6%)

Steroid use	None	216 (55.0%)	27 (96.4%)
	Dexamethasone	166 (42.2%)	1 (3.6%)
	Prednisolone	11 (2.8%)	0 (0.0%)

Steroid dose (mg/day)	Dexamethasone	6 (4–24)	6 (6–6)
	Prednisolone	40 (20–40)	–

Outcome	Complete treatment at hospitel	325 (82.7%)	27 (96.4%)
	Hospitel transfer	68 (17.3%)	1 (3.6%)

Final outcome	Discharge, alive	391 (99.5%)	28 (100%)
	Death at the hospitel	2 (0.5%)	0 (0%)

Adverse drug reactions		Diarrhea 1 (0.3%), maculopapular rash 1 (0.3%), nausea 1 (0.3%), palpitation 1 (0.3%)	Vomiting 2 (7.1%)

Data were presented as frequency (percent) except for the steroid dose, which was presented as median (minimum–maximum).

**Table 5:** Factors associated with hospitel transfer among 393 adult patients.

Characteristics		Complete treatment at hospitel (*n* = 325)	Transfer to hospitel (*n* = 68)	Univariate analysis OR (95% CI), *p* value	Multivariate analysis OR (95% CI), *p* value
Age		45 (18–72)	54 (20–73)	1.06 (1.03–1.08), <0.001^*∗*^	1.06 (1.02–1.10), 0.002^*∗∗*^
Gender (male)		114 (35.1%)	33 (48.5%)	1.74 (1.03–2.96), 0.038^*∗*^	1.10 (0.49–2.50), 0.813
Body mass index (kg/m^2^)		24.2 (15.8–38.3)	25.8 (15.9–33.1)	1.07 (1.00–1.14), 0.046^*∗*^	1.03 (0.93–1.15), 0.539

Underlying diseases	Hypertension	49 (15.1%)	17 (25.0%)	1.88 (1.00–3.52), 0.049^*∗*^	0.62 (0.20–1.91), 0.402
	Dyslipidemia	22 (6.8%)	8 (11.8%)	1.84 (0.78–4.32), 0.164	
	Diabetes mellitus	14 (4.3%)	10 (14.7%)	3.83 (1.62–9.04), 0.002^*∗*^	3.46 (0.66–18.16), 0.143
	Allergy	17 (5.2%)	1 (1.5%)	0.27 (0.04–2.07), 0.208	
	Musculoskeletal disease	6 (1.8%)	3 (4.4%)	2.45 (0.60–10.06), 0.213	
	Digestive disease	7 (2.2%)	1 (1.5%)	0.68 (0.08–5.60), 0.718	
	HIV	5 (1.5%)	2 (2.9%)	1.94 (0.37–10.21), 0.434	
	Neurological disease	8 (2.5%)	2 (2.9%)	1.20 (0.25–5.78), 0.820	
	Liver disease	4 (1.2%)	1 (1.5%)	1.20 (0.13–10.89), 0.873	
	Malignancy	2 (0.6%)	2 (2.9%)	4.89 (0.68–35.37), 0.116	
	Heart disease	5 (1.5%)	1 (1.5%)	0.96 (0.11–8.31), 0.967	
	Psychological disease	4 (1.2%)	2 (2.9%)	2.43 (0.44–13.55), 0.311	
	Lung disease	3 (0.9%)	1 (1.5%)	1.60 (0.16–15.64), 0.685	
	Thalassemia	4 (1.2%)	1 (1.5%)	1.20 (0.13–10.89), 0.873	
	Thyroid disease^a^	4 (1.2%)	0 (0.0%)	–	

Day of illness		6 (1–17)	6 (1–11)	0.97 (0.89–1.06), 0.523	

Symptoms	Fever	118 (36.3%)	40 (58.8%)	2.51 (1.47–4.27), 0.001^*∗*^	1.16 (0.48–2.81), 0.747
	Cough	201 (61.8%)	46 (67.6%)	1.29 (0.74–2.25), 0.369	
	Oxygen desaturation	84 (25.8%)	47 (69.1%)	6.42 (3.63–11.37), <0.001^*∗*^	1.90 (0.83–4.35), 0.130
	Dyspnea	72 (22.2%)	30 (44.1%)	2.77 (1.61–4.79), <0.001^*∗*^	2.84 (1.25–6.44), 0.013^*∗∗*^
	Sore throat	75 (23.1%)	11 (16.2%)	0.64 (0.32–1.29), 0.214	
	Rhinorrhea	45 (13.8%)	13 (19.1%)	1.47 (0.74–2.91), 0.267	
	Productive sputum	28 (8.6%)	6 (8.8%)	1.03 (0.41–2.58), 0.956	
	Myalgia	22 (6.8%)	6 (8.8%)	1.33 (0.52–3.42), 0.550	
	Diarrhea	17 (5.2%)	5 (7.4%)	1.44 (0.51–4.04), 0.491	
	Anosmia	16 (4.9%)	3 (4.4%)	0.89 (0.25–3.15), 0.858	
	Headache	14 (4.3%)	4 (5.9%)	1.39 (0.44–4.36), 0.574	
	Dizziness^a^	12 (3.7%)	0 (0.0%)	–	
	Malaise	6 (1.8%)	3 (4.4%)	2.45 (0.60–10.06), 0.213	
	Nasal congestion^a^	8 (2.5%)	0 (0.0%)	–	
	Loss of taste	1 (0.3%)	5 (7.4%)	25.71 (2.95–223.85), 0.003^*∗*^	107.63 (1.24–9337.39), 0.040^*∗∗*^
	Chest pain	2 (0.6%)	2 (2.9%)	4.89 (0.68–35.37), 0.116	
	Nausea	2 (0.6%)	2 (2.9%)	4.89 (0.68–35.37), 0.116	

Chest X-ray category^a^	1	5 (1.5%)	0 (0.0%)	–	
	2	7 (2.2%)	0 (0.0%)	–	
	C	4 (1.2%)	0 (0.0%)	–	
	3	125 (38.5%)	28 (41.2%)	–	
	4	97 (29.8%)	15 (22.1%)	–	
	5	87 (26.8%)	25 (36.8%)	–	

Indications of favipiravir^a^	Asymptomatic/mild symptom without risk factors	5 (1.5%)	0 (0.0%)	–	
	Mild symptom with risk factors	8 (2.5%)	0 (0.0%)	–	
	Pneumonia	217 (66.8%)	21 (30.9%)	–	
	Pneumonia with hypoxia	70 (21.5%)	44 (64.7%)	–	
	Pneumonia with progression of infiltrates	25 (7.7%)	3 (4.4%)	–	

Start favipiravir on admission		169 (52.0%)	32 (47.1%)	0.82 (0.49–1.38), 0.459	
Extended duration of favipiravir		42 (12.9%)	55 (80.9%)	28.51 (14.36–56.60), <0.001^*∗*^	16.91 (7.29–39.24), <0.001^*∗∗*^
Steroid use		113 (34.8%)	64 (94.1%)	30.02 (10.66–84.56), <0.001^*∗*^	12.56 (3.65–43.18), <0.001^*∗∗*^

^a^Logistic regression was not analyzed as no patient was transferred to the hospitel. ^*∗*^*P* < 0.01. ^*∗∗*^*P* < 0.05. Data were presented as frequency (percent) except for age, body mass index, and day of illness, which were presented as median (minimum–maximum). OR, odds ratio; CI, confidence interval.

## Data Availability

The data used to support the findings of this study are available from the corresponding author upon request.
